# LA-MRSA CC398 in Dairy Cattle and Veal Calf Farms Indicates Spillover From Pig Production

**DOI:** 10.3389/fmicb.2019.02733

**Published:** 2019-11-26

**Authors:** Julie E. Hansen, Troels Ronco, Marc Stegger, Raphael N. Sieber, Mette E. Fertner, Henrik L. Martin, Michael Farre, Nils Toft, Anders R. Larsen, Karl Pedersen

**Affiliations:** ^1^National Veterinary Institute, Technical University of Denmark, Lyngby, Denmark; ^2^Department of Bacteria, Parasites and Fungi, Statens Serum Institut, Copenhagen, Denmark; ^3^SEGES P/S, Livestock Innovation, Aarhus, Denmark; ^4^National Veterinary Institute, Uppsala, Sweden

**Keywords:** bulk tank milk, veal calves, cattle, methicillin resistant *Staphylococcus aureus*, CC398, *mecC*, WGS

## Abstract

The possible spillover from pigs into other production animals incites concern for unresolved reservoirs of human exposure. The present investigation was therefore initiated, to elucidate if Danish veal and dairy farms constitute a reservoir of livestock-associated methicillin-resistant *Staphylococcus aureus* (LA-MRSA) CC398 and to potentially identify the source of introduction. We collected nasal swab samples from 17 Danish veal farms, 2 slaughterhouses, and received bulk tank milk samples from 286 dairy farms. All samples were analyzed by culturing and screening on MRSA selective plates and presumed MRSA was verified by MALDI-TOF and PCR. MRSA isolates were subjected to *spa* typing and whole-genome sequencing. LA-MRSA was found on two veal farms in one and three calves, respectively, with subsequent follow-up samples found negative. Eight of 286 dairy farms (2.8%) were found LA-MRSA positive and follow-up samples, from five farms showed intermittent detection of LA-MRSA. The *spa* types, t034 and t011, were the most common while a single isolate from a dairy farm belonged to *spa* type t843 associated to *mecC*-MRSA CC130 and is the first report of *mecC*-MRSA in the Danish dairy production. A phylogenetic analysis showed that some of the isolates grouped within or close to the dominant Danish pig clusters, suggesting spillover into cattle farms. Other isolates clustered outside the dominant pig clusters suggesting that other routes of introduction cannot be excluded. Results of the investigation indicated a contamination of veal farms while some dairy farms seemed to be a permanent reservoir. Thus, Danish cattle represent a low prevalence reservoir of LA-MRSA CC398, which at present, is not of major human health concern.

## Introduction

The presence of livestock-associated methicillin-resistant *Staphylococcus aureus* (LA-MRSA) clonal complex (CC)398 has been reported in a wide range of different food animals from multiple European countries (Cuny et al., 2010). Like many other European countries, Denmark has experienced a rapid increase of LA-MRSA CC398 in its pig production. A baseline study conducted by the European Food Safety Authority (EFSA) in 2008 found a prevalence of 3.5% in Danish production holdings with breeding pigs and 0% in breeding holdings (EFSA, 2009). However, in 2016, an official survey found a prevalence of LA-MRSA in randomly selected finisher production herds of 88%, an increase from 68% in 2014 (DANMAP, 2014, 2016). This development has gained considerable attention in Denmark due to the concurrent increase of humans tested positive for LA-MRSA CC398 (DANMAP, 2016). As a result, concerns were raised about the possible spillover of LA-MRSA CC398 from the pig production into other livestock, such as dairy cattle and veal calf production. LA-MRSA CC398 isolates have been reported in different bovine reservoirs from several countries and transmission of LA-MRSA to personnel has also been described (Juhász-Kaszanyitzky et al., 2007; Graveland et al., 2010; Fessler et al., 2012; Vandendriessche et al., 2013; Van Cleef et al., 2015).

On the basis of the rapid dissemination of LA-MRSA CC398 throughout the Danish pig production, and the expansion of the animal host range found in other European countries, this study was initiated with the purpose of investigating if Danish veal and dairy farms constitute a reservoir of LA-MRSA CC398 and to assess whether pigs could be a potential source of introduction. For dairy farms, a single investigation has so far been performed in Denmark. In 2012, 4/219 (1.8%) bulk tank milk (BTM) samples were found LA-MRSA positive (DANMAP, 2012). However, given the dramatic increase in the prevalence of LA-MRSA CC398 seen in pigs, new information about the level of LA-MRSA CC398 in Danish dairy farms is relevant.

The possible association with the Danish pig production of LA-MRSA CC398 isolated from other animals than pigs can be established with WGS and phylogenetic analysis based on single-nucleotide polymorphisms (SNPs). In Denmark, three clonal lineages of LA-MRSA, L1–L3 are known to dominate in the pig production (Sieber et al., 2018). Certain genomic markers, such as *tet*(M), *tet*(K), and *czrC*, are indicators of pig-association while genes related to the human immune evasion cluster (IEC) are typically found in isolates of primary human origin (McCarthy et al., 2012; Price et al., 2012). The staphylococcal cassette chromosome *mec* (SCC*mec*), sub-type Vc(5C2&5), and the *spa* types t011 and t034, have previously been identified widely among CC398 LA-MRSA isolates from pigs but also among humans and cattle (Price et al., 2012; Paterson et al., 2013; Larsen et al., 2015, 2016). In ruminants, two variants of the von Willebrand binding protein encoded by the gene *vwb* have been shown to increase virulence (Viana et al., 2010). The presence or absence of these marker genes can aid in the identification of the origin of a given CC398 LA-MRSA isolate.

## Materials and Methods

### Sample Collection

#### Veal Calves

Samples from veal calves were collected in 2015 at slaughter as well as on farms. Using swabs (ESwab, Copan, Corona, CA, United States), samples from the nose, the groin, and the perianal region from 93 calves were collected at slaughter during two sampling rounds at an abattoir. A minimum of five animals per farm was obtained, and the 93 calves (8–12 months old) originated from 15 different rosé veal calf holdings who received their calves from 45 different dairy cattle farms.

In addition, nasal swabs from 620 veal calves, 2–4 months old, were collected on farm from 17 different rosé veal calf holdings, a minimum of 25 samples per farm to achieve at least the same within-farm sensitivity as in the Danish screening of pig farms published in DANMAP (2014). The farms were selected to geographically represent various regions of Denmark, and all farm holdings included in this study were large production holdings with 300–2000 veal calves on site.

#### Dairy Farms

Bulk tank milk samples were obtained from various dairy farms during multiple collection rounds. In 2014, samples were obtained from 50 dairy farms randomly selected among the Danish milk producers. None of these farms were connected. Follow-up samples were collected at 10 different time points over the following 9 months, from five dairy farms associated by a common owner, from which one farm was included in the initial screening of 50 farms. In 2015, a total of 236 samples representing 236 unconnected farms were received for screening, collected on farm by the dairy company Arla, and submitted by the diagnostic laboratory, Eurofins as previously described (Ronco et al., 2018).

### Sample Processing and MRSA Identification

Swabs were enriched in 5 ml Mueller–Hinton broth supplemented with 6.5% NaCl for 18–24 h of incubation at 37°C whereafter a 10 μl loopful of broth was streaked on selective Brilliance MRSA2 agar plates (Thermo Fisher Scientific, Oxoid, Basingstoke, United Kingdom) and incubated for 18–24 h at 37°C. From plates displaying colonies suspect of MRSA, one presumptive MRSA colony was sub-cultivated onto blood agar. Isolates were identified as *S. aureus* by MALDI-TOF (Bruker, Bremen, Germany). Multiplex PCR was performed to detect *mecA* and *nuc* genes (Maes et al., 2002) and an additional duplex PCR detecting *mecC* (García-Álvarez et al., 2011) was performed, if *mecA* negative. Such confirmed MRSA isolates were subsequently stored at −80°C until further analysis.

One milliliter of each BTM sample was enriched 18–24 h at 37°C in 9 ml Mueller–Hinton broth supplemented with 6.5% NaCl, and subsequently streaked onto Brilliance MRSA 2 agar (Oxoid, Basingstoke, United Kingdom). Presumptive MRSA colonies were treated as stated above. *spa* typing of all MRSA isolates was performed as previously described (Mellmann et al., 2006; Stegger et al., 2012).

### Strain Collection for Whole-Genome Sequencing

A total of 19 MRSA isolates from the screening of dairy (*n* = 15) and veal calf (*n* = 4) farms were available for whole-genome sequencing. In 2016, an aseptic foremilk sample was collected from a Danish dairy cow with clinical mastitis according to the National Mastitis Council’s guidelines, from which LA-MRSA strain Sa52 was derived (Ronco et al., 2017). This mastitis isolate was included in the genomic characterization and phylogenetic analysis. Sequences from isolates of LA-MRSA CC398 from Danish pigs (*n* = 183) previously described by Sieber et al. (2018) were included for comparison as were sequences of 88 international CC398 isolates previously described by Price et al. (2012) as a reference collection. These LA-MRSA (*n* = 48) and MSSA (*n* = 40) isolates were sampled from both humans (*n* = 25) and livestock (*n* = 63) in 19 different countries located on four continents. (The livestock samples included strains from live animals, meat samples, and environmental contamination.)

### Strain Preparation and Whole-Genome Sequencing

To extract DNA from the 19 MRSA isolates (veal calves = 4 and BTM = 15) the DNeasy Blood and Tissue kit (Qiagen, Hilden, Germany) was used according to the manufacturer’s instruction, except that a pretreatment with lysostaphin was applied before extraction as described by Strube et al. (2018). The library preparation was performed using an Illumina Nextera XT kit (Illumina Inc., San Diego, CA, United States) and run on Illumina’s NextSeq instrument for 150-bp paired-end sequencing. The raw reads for the single clinical mastitis isolate Sa52 can be found in the Sequence Read Archive (SRA) under the accession number SRX3279848 whereas raw reads from the veal calf and BTM isolates have be deposited under the study accession no SRP129828. Whole-genome sequencing data for the international reference collection (Price et al., 2012) were obtained from the SRA via BioProject accession number PRJNA274898. The Danish pig strain sequences were obtained from Sieber et al. (2018) via BioProject accession number PRJEB25608 in the European Nucleotide Archive (ENA).

### Single-Nucleotide Polymorphism Calling and Phylogenetic Analysis

Using NASP (Sahl et al., 2016), identification of SNP variants was performed using the GATK UnifiedGenotyper with filtering set to remove positions with less than 10-fold coverage and 90% unambiguous variant calls after positions within duplicated regions of the reference sequence (*S. aureus* S0385, GenBank accession number AM990992) were removed using NUCmer. Purging of the 123 kb recombinant region of the ST398ST9 hybrids was performed prior to phylogenetic reconstruction using FastTree version 2.1.5 as implemented in Geneious version 11.0.2 (Biomatters, Auckland, New Zealand) and rooted according to Price et al. (2012).

### Genomic Characterization of Isolates

Multilocus sequence types (MLST) were identified using the PubMLST database (Jolley and Maiden, 2010) and the presence of resistance determinants was assessed using raw sequence reads in Mykrobe Predictor version 0.4.3 (Bradley et al., 2015). Using the same software, the genotype of additional genes of interest was determined with results filtered for >80% coverage and >5 median depth. The reference sequences were obtained from NCBI genomes with accession number DQ530361 for Sa3int, *sak*, *scn*, and *sea*, BA000018.3 for *sep*, KF593809.1 for the cadmium-zinc resistance gene *czrC*, NC_013450 for genes SAAV_2008 and SAAV_2009 associated with virulence in avian hosts; and HM211303, HM228919, and HM228920 for the ruminant-specific *vwb* virulence genes. Finally, the online tool SCC*mec*Finder 1.2 (Kaya et al., 2018) was applied for SCC*mec* typing using a nucleotide identity threshold of 80% and a minimum coverage of the query sequence of 60%.

## Results

### Occurrence of MRSA in Veal Calves and Bulk Tank Milk

#### Veal Calves

No MRSA was found in any of the samples taken from calves at the slaughterhouse, and no new information of optimal anatomical sampling site was obtained. MRSA was found in samples from 2 of the 17 (11.8%) farms in one and three calves, respectively. All four isolates were typed as *spa* type t034, which is known to belong to LA-MRSA CC398. In a follow-up investigation (11/2 month later), the four calves and their pen mates were negative.

#### Dairy Farms

A total of 286 BTM samples were screened for the presence of MRSA and 8 samples were found to be MRSA positive corresponding to 3% of the farms. Seven of the eight isolates were *mecA* positive and of *spa* type t034 belonging to CC398 (Table 1) while a single isolate was of *spa* type t843 associated to *mecC*-MRSA CC130. The *mecC*-MRSA was excluded from the phylogenetic analysis and further genomic characterization.

**TABLE 1:** Presence of *spa* types, SCC*mec* types, and resistance genes among MRSA isolates.

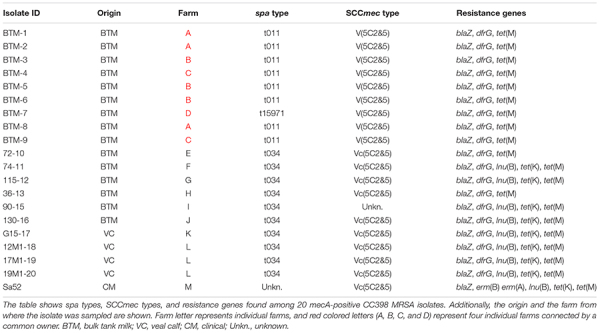

One positive sample was obtained from a farm where the owner had four additional dairy cattle farms, where farm workers and animals were moved between farms. Hence, follow up samples were taken at all five farms. Samples from the linked farms were collected approximately monthly during a period of 9 months, resulting in a total of 40 samples, which were investigated for MRSA. Ten of these samples contained LA-MRSA, with three farms intermittently found positive and two farms never found positive. All 10 isolates belonged to CC398: Nine had *spa* type t011 (repeat succession: 08-16-02-25-34-24-25) and a single isolate had a new *spa* type, t15971 (repeat succession: 08-16-02-25-34-24-25-02-25-34-24-25). Nine of the detected isolates were available for sequencing and further genetic characterization.

### Genomic Characterization of the Isolates

Isolates from the clinical mastitis case (*n* = 1), veal calves (*n* = 4), and BTM (*n* = 15) were characterized on the basis of genomic identification of resistance genes and additional genes associated to different host. None of the isolates were found to carry any of the human-related IEC genes (Sa3int, *sak*, *scn*, *sea*, and *sep*), genes related to ruminant (*vwb*) or avian host-specificity SAAV_2008 and SAAV_2009. All 20 isolates were found to carry *mecA*, *tet*(M), and *blaZ*, whereas *dfrG* was detected in 19/20 (95%) of the isolates, but absent in the mastitis strain Sa52 (Table 1). Strain Sa52, the four isolates from veal calves and six of the BTM isolates were found to carry the gene encoding cadmium-zinc resistance, *czrC*; however, the nine isolates originating from the four common owner dairy farms were found not to carry neither *czrC* nor *tet*(K). The *lnu*(B) gene was found in 9/20 (45%) isolates and two genes encoding erythromycin resistance, *erm*(A) and *erm*(B) were detected only in the mastitis isolate (Table 1). In addition, all eight bovine t011 isolates and the single t15971 isolate, carried the SCC*mec* element V(5C2&5). In contrast, the majority (9/10) of the bovine t034 isolates and a single isolate of unknown *spa* type carried the Vc(5C2&5) element. The V(5C2&5) element was identified only among the BTM isolates and with approximately 72% coverage of the query sequence, whereas the Vc(5C2&5) element was observed among isolates from BTM, veal calves, and clinical mastitis, with ≥90% coverage of the query sequence (Table 1).

### Phylogenetic Analysis

**FIGURE 1:**
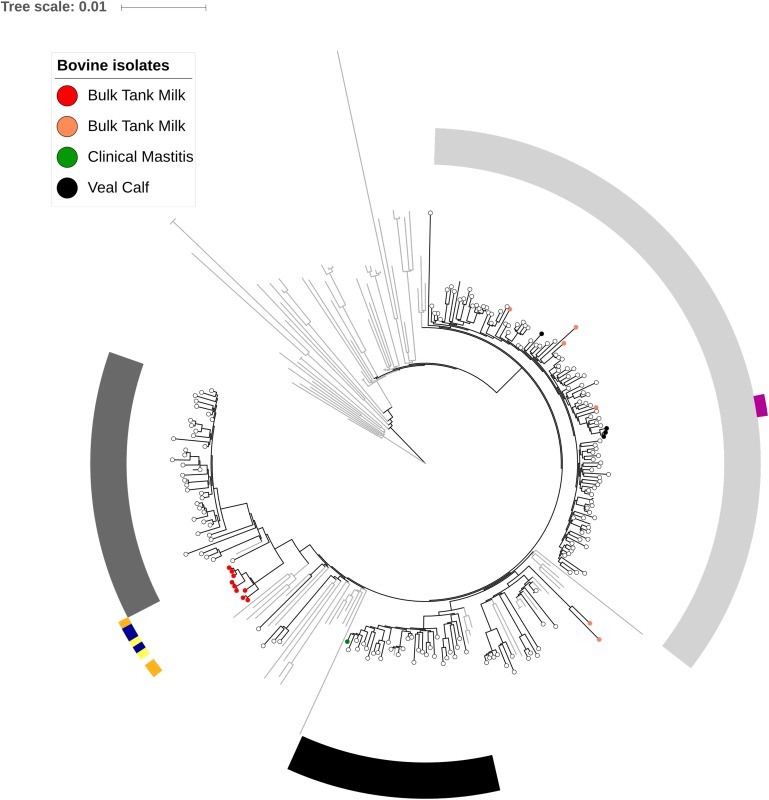
Phylogenetic relationship of LA-MRSA CC398 from bulk tank milk, veal calves, pigs, and from a worldwide reference collection. The phylogeny includes 20 LA-MRSA isolates from multiple bovine sources. Bulk tank milk is divided in red dots which correspond to isolates from four farms with a common owner and orange dots correspond to unrelated farms. Isolates collected from the same farm are marked with strips of identical color. Orange, Farm A; blue, Farm B; yellow, Farm C; and purple, Farm L. A total of 183 isolates related to Danish pigs from 2014 (Sieber et al., 2018) are seen as black open circle and 89 international isolates included as reference phylogeny from Price et al. (2012) are seen as gray nodes. Dominating pig lineages are seen in L1 = dark gray, L2 = black, and L3 = light gray strips. The tree has been rooted according to Price et al. (2012).

Based on the phylogenetic relationship seen in Figure 1, 9/20 bovine isolates were found to cluster within the three dominating lineages, L1–L3, present in Danish pigs. The nine BTM isolates from the three farms with a common owner clustered close to L1; however, no Danish pig isolates were found in this BTM clade. All but three of the remaining isolates belonged to the L3-clade, whereas strain Sa52 was in the L2-clade and two BTM isolates were placed outside of the dominant pig lineages.

## Discussion

The nostrils were chosen as sampling location throughout on the farm samplings, as it was assessed to provide the most comparable results in respect of previous reported findings in the literature. We found LA-MRSA CC398 in 2 of 17 Danish veal farms. In the two positive farms, however, no evidence of persistent LA-MRSA CC398 colonization in veal calves was observed. In Danish dairy cattle, we found LA-MRSA CC398 in BTM from 8 of 286 dairy farms. Repeated sampling of BTM in one of the positive farms and four contact farms showed intermittent detection of LA-MRSA CC398. Based on these findings, we conclude that LA-MRSA CC398 was present in the Danish dairy and veal calf production, however, in considerably lower frequencies than observed in the Danish pig production (DANMAP, 2016). A similar low prevalence of LA-MRSA has been observed among bovine mastitis isolates (*n* = 120) collected from eight different countries across the world and among BTM isolates (*n* = 486) from southern Italy (Parisi et al., 2016; Monistero et al., 2018). Furthermore, a recent study from Germany based on quarter milk samples suggested that LA-MRSA is not common in milk from dairy cows. The authors found *S. aureus* in 372 out of 14,924 milk samples, of which 10 were LA-MRSA, all CC398 (Kadlec et al., 2019). Prevalence studies carried out in other European countries, such as Netherlands and Germany (Bos et al., 2012; Tenhagen et al., 2014), have found a high prevalence of LA-MRSA CC398 in veal calf farming. Differences in management factors, production types (rosé veal vs. white veal), and usage of antimicrobials might influence the differences seen in prevalence compared to Denmark.

In the present study, the most prevalent *spa* types were t034 and t011, which is in accordance with previous findings, where LA-MRSA CC398 t011 was found in Danish retail beef, and t011 and t034 in BTM (Agersø et al., 2012; DANMAP, 2012). Both *spa* types are recognized as the most common *spa* types in the Danish pig production (DANMAP, 2014) but can also be found in humans, mink, or horses (DANMAP, 2016; Islam et al., 2017; Hansen, 2018). A new *spa* type, t15971, was detected in BTM, which could have resulted from a single genetic event in t011 with the duplication of five repeats 02-25-34-24-25 (Table 1).

The identified *spa* types and the low prevalence of LA-MRSA CC398 positive veal and dairy farms could indicate spillover from the pig production. Tavakol et al. (2012) previously provided evidence of transmission of LA-MRSA CC398 from pigs to cattle, and Locatelli et al. (2016) illustrated an exposure–response relationship between the LA-MRSA positive status of dairy farms and the number of surrounding pig farms. Based on the phylogenetic analysis (Figure 1), it is evident that some isolates found in the different bovine sources were of the same lineages as those found in the pig production. This indicates that the presence of LA-MRSA CC398, to some extent, is a result of spillover from pigs into the Danish dairy and veal calf production. It may also be explained by the presence of a common source but pigs are by far the largest reservoir of LA-MRSA and therefore, other sources seem less likely to be the case. Of note, even though the ruminant host-specific *vwb* genes were not found in bovine isolates, they were found in 6% (11/183) of Danish pig isolates, 10 of which belonging to lineage L2.

Notably, the 20 LA-MRSA isolates from multiple bovine sources, included in Figure 1, were sampled from 13 farms. Nine of these farms were located approximately 1 km from a pig farm and a worker on one farm also worked in the pig production. Combined, this implied that a high proportion (10/13) of farms, from where these 20 isolates originated, could potentially be related to pig holdings.

Interestingly, the four veal calf isolates and four BTM isolates found in L3 had identical resistance patterns, whereas the two BTM isolates clustering on their own did not carry the additional tetracycline resistance gene, *tet*(K), characteristic for pig-associated LA-MRSA CC398. The loss of *tet*(K) has previously been observed in isolates removed from the selective pressure of tetracycline (Larsen, personal Communication). It is possible that these two isolates did not come directly from pigs but have been introduced into the dairy farms some time ago after a period of absence from the farm environment, while occupying another host, e.g., humans. Human introduction in pig farms is not uncommon as observed in Norway (Grøntvedt et al., 2016) and the likely introduction of an MRSA strain into a dairy herd with subsequent cases of mastitis has recently been described from Sweden (Unnerstad et al., 2018). However, the absence of human and bovine host-associated genetic elements and the presence of *tet*(M) and *dfrG* in the two isolates indicate pigs as the most probable origin of the strains, as these two genes are indicators of association to the pig-clade (McCarthy et al., 2012). Since pigs and cattle is not kept in the same facilities or transported on the same trucks, it may be suggested that LA-MRSA CC398 have been introduced into the veal calf and dairy farms via colonized farm workers, but this needs to be further investigated. The strain from clinical mastitis Sa52 was the only isolate which harbored the erythromycin resistance genes, *erm*(A) and *erm*(B), and lacked *dfrG*. Erythromycin resistance is more commonly found in pig isolates in L2 compared to L1 and L3 (Sieber et al., 2018), which is in agreement with the location of Sa52 within L2 as the only bovine isolate.

The nine BTM isolates from the four connected dairy farms with a common owner were found to be phylogenetically closely related, and contained identical accessory genes suggesting a single introduction and spread. Interestingly, these nine BTM isolates were located outside the dominant pig lineages L1, L2, and L3 (Figure 1) and all carried the type V(5C2&5) SCC*mec* element (Table 1). In addition, none of the nine BTM isolates carried *tet*(K) or *czrC*, which have been associated with pig-adaptation (Table 1). This may suggest another route of introduction, possibly human. The lack of *czr*C could be a result of absence of selective pressure caused by zinc as a feed additive for pigs triggered the loss of the cadmium-zinc resistance gene (Price et al., 2012; Larsen et al., 2016). In contrast, nine of the isolates that clustered within the dominant pig lineages (Figure 1) carried the *tet*(K) gene and eight of these also carried the Vc(5C2&5) SCC*mec* (Table 1). Larsen et al. (2016) found that isolates carrying the Vc(5C2&5) element also harbored *tet*(K) in a higher proportion than isolates with other SCC*mec* elements. Previously, the Vc(5C2&5) SCC*mec element* has also been found among CC398 LA-MRSA from pigs, cattle, and humans (Price et al., 2012; Paterson et al., 2013; Larsen et al., 2016). Therefore, further investigation of the genetic content related to isolates within the dominant pig lineages and those outside could possibly provide valuable information in regard to their origin and dissemination. This study is limited by solely investigating the resistance genotype of the LA-MRSA CC398 isolates. It is therefore not known if the detected bovine isolates displayed the same resistance phenotype, it might be that not all detected genes are expressed. Previous studies (Zankari et al., 2012; Larsen et al., 2017) have shown that there is a high correspondence between the resistance geno- and phenotype and thus, we did not find it necessary to carry out phenotyping of the LA-MRSA CC398 isolates analyzed in present study.

The intermittent detection of LA-MRSA isolates from the three farms indicates low prevalence close to the detection limit. This could be a result of subclinical mastitis in the farm as observed in Belgium (Vanderhaeghen et al., 2010), and low within-farm prevalence of intra-mammary infections in dairy cows caused by LA-MRSA CC398 has previously been reported (Spohr et al., 2011; Luini et al., 2015). The first confirmed clinical mastitis LA-MRSA isolate from Denmark was included in the present study and shows that LA-MRSA CC398 is able to cause mastitis in Danish dairy farms. However, in concordance to the present study, Ronco et al. (2018) showed low prevalence of LA-MRSA ST398 isolates among Danish *S. aureus* isolates from BTM (0/94 isolates) and cases of clinical mastitis (1/63 isolates). In 2012, a study using the same methodology as the present study also found a similar low prevalence and intermittent detection of LA-MRSA CC398 in Danish BTM (DANMAP, 2012).

However, the first *mecC*-MRSA isolate from a Danish dairy farm was identified in the present study. *mecC*-MRSA was first detected in isolates from bovine milk samples (García-Álvarez et al., 2011) and a Danish study from Petersen et al. (2013) concluded that ruminants may be healthy carriers of CC130 *mecC-*MRSA and documented transmission in a single case from a cow to its owner (Harrison et al., 2013). The detection of a single CC130 *mecC*-MRSA in BTM in the present study indicates that *mecC*-MRSA is not, at this point, widespread in the cattle production and far from the success that LA-MRSA CC398 has in pig production.

The present study in veal calf holdings is based on a relatively low number of screened farms. We found that LA-MRSA CC398 is detectable in the Danish cattle production, but more farms should be included to determine the prevalence properly. We expected a low prevalence of LA-MRSA-positive farms based on the results of the samples collected at the abattoir, but we had anticipated a higher within-farm prevalence based on previous experiences from LA-MRSA-positive pig holdings (Hansen, 2018). This seems not to be the case in cattle. Thus, in future screenings, the number of animals at cattle holdings should be increased in order to increase the sensitivity of detecting LA-MRSA.

## Conclusion

Results obtained in this study show LA-MRSA CC398 to be present in Danish dairy and veal calf farms. The phylogenetic analyses suggest some spillover from pig production into cattle production since some of the isolates clustered inside the main clonal lineages from pigs. However, other isolates clustered outside these main lineages. These isolates may still represent spillover from pigs but the existence of another source cannot be excluded. Based on the genomic characterization, it seems that the veal farms and some dairy farms are merely transiently contaminated, while persistent low presence of LA-MRSA CC398 in some dairy farms was observed. Of notice, *mecC*-MRSA was for the first time identified in a Danish dairy farm. The few detected cases in dairy farms and veal calf farms indicate that the cattle production does not represent a substantial reservoir of LA-MRSA CC398 of concern for human health concern at this point. However, lessons from the spread in veal calves and dairy cattle in the Netherlands as well as the Danish experience with CC398 in pigs may emphasize the need to routinely conduct investigations to survey potential changes.

## Data Availability Statement

The whole-genome sequence data generated in this study have been submitted to the European Nucleotide Archive under BioProject accession number PRJNA430150.

## Ethics Statement

Nasal and skin swab sampling from calves was carried out in accordance with Danish legislation. No ethical approval from the National Animal Experimentation Council or from the Institutional Ethics Committee, The Animal Welfare Body at DTU National Veterinary Institute, was required. Samples were collected by staff from SEGES with consent from the owners of the animals.

## Author Contributions

JH coordinated the sampling and culture of samples, prepared the DNA for sequencing, analyzed the sequence data, and drafted the manuscript. TR assisted in analyzing sequence data and participated in preparing the manuscript. MS was responsible for the sequencing and supported the analysis of sequence data. RS connected the sequence data with previous sequences from pigs and humans. MEF assisted in sampling veal calves and analyzing samples. HM planned the sampling of calves and performed the sampling. MF was responsible for sampling of BTM sampling. NT was overall responsible for the project plan and assisted in drafting the manuscript. AL assisted with the planning of the study, sequencing, and drafting of the manuscript. KP assisted in planning and coordinating the study, coordinating the sample flow and laboratory work, analyzing the data, and drafting the manuscript. All authors read and approved the final manuscript.

## Conflict of Interest

The authors declare that the research was conducted in the absence of any commercial or financial relationships that could be construed as a potential conflict of interest.
